# Reliability of 3D Stereophotogrammetry for Measuring Postoperative Facial Swelling

**DOI:** 10.3390/jcm11237137

**Published:** 2022-11-30

**Authors:** Margje B. Buitenhuis, Reinoud J. Klijn, Antoine J. W. P. Rosenberg, Caroline M. Speksnijder

**Affiliations:** 1 Department of Oral and Maxillofacial Surgery, University Medical Center Utrecht, Utrecht University, 3584 CX Utrecht, The Netherlands; 2 Department of Oral and Maxillofacial Surgery, Medisch Spectrum Twente, 7512 KZ Enschede, The Netherlands

**Keywords:** three-dimensional, bilateral sagittal split osteotomy, reliability, stereophotogrammetry, swelling

## Abstract

This study aimed to determine the reliability of three-dimensional (3D) stereophotogrammetry as a measurement instrument for evaluating soft tissue changes in the head and neck area. Twelve patients received a bilateral sagittal split osteotomy (BSSO). Test and retest 3D photographs were captured within the first three postoperative weeks, and a reference 3D photograph was capture at three months postoperatively. Distance measurements, mean and root mean square of the distance map, and volume differences were obtained. Reliability of these parameters was assessed by intraclass correlation coefficients (ICCs), standard error of measurement (SEM), and smallest detectable change (SDC). All distance measurements had an ICC > 0.91, and the distance map parameters and volume differences showed ICCs > 0.89. The neck region presented the largest SEMs (5.09 mL) and SDC (14.1 mL) for the volume difference. In conclusion, 3D stereophotogrammetry is reliable for distance and volume measurements of soft tissues in patients after a BSSO advancement.

## 1. Introduction

Every patient in orthognathic surgery encounters postoperative swelling. When postoperative swelling disappears over time, patients can finally observe surgical soft tissue results. Although a rapid reduction in swelling is common, assessment and quantification in the early period is clinically relevant and beneficial for research purposes [1,2]. The extent and duration of postoperative swelling provides information to accurately administer and monitor steroidal or nonsteroidal anti-inflammatory drugs to accelerate the reduction of swelling [1,3,4]. Other methods to prevent and reduce postoperative swelling are cooling, Kinesio taping, manual lymphatic drainage, and low-level laser therapy, mostly recommended for optimal patient comfort [1,5,6,7,8].

In order to assess and quantify edema and postoperative swelling, multiple methods have been proposed [1,9]. Gold-standard tape measurements are inexpensive, simple, and non-invasive. However, this technique has been replaced by newer non-contact methods, such as optical three-dimensional (3D) scanners [3]. Three-dimensional stereophotogrammetry has increasingly been used in orthognathic surgery planning and evaluation. However, the feasibility of 3D stereophotogrammetry for measuring soft tissue changes caused by swelling has only been evaluated to a small extent in orthognathic patients. Earlier studies showed that the 3dMD stereophotogrammetry system can accurately measure artificial cheek swelling in healthy subjects [10,11]. Application of 3D stereophotogrammetry in predicting postoperative facial appearance after Le Fort I osteotomy presented acceptable reproducibility, with intraclass correlation coefficients (ICCs) ranging from 0.87 to 0.99 [12]. Three-dimensional stereophotogrammetry volumetric analysis strongly correlated with tape measurements for surgically assisted rapid maxillary expansion (SARME), with correlations varying from 0.98 to 0.99 [3]. Both studies focused on edema in the midface, while postoperative swelling after orthognathic surgery mostly occurs in all areas of the face and neck. The most common orthognathic procedure is the bilateral sagittal split osteotomy (BSSO). Postoperative swelling after a BSSO is expected in the lower face and neck area [4]. The reliability of 3D stereophotogrammetry measurements in these areas is not yet determined. Reliability is defined as the extent to which measurements do not change for repeated measurements [13].

Therefore, the goal of this study is to determine the reliability of 3D stereophotogrammetry as a measurement instrument for evaluating soft tissue changes in the head and neck area in patients receiving BSSO advancement.

## 2. Materials and Methods

### 2.1. Study Design

This prospective cohort study took place at the University Medical Center Utrecht (UMCU). Approval for this study was retrieved from the medical ethical committee of the UMCU (METC protocol number 20-780). Written informed consent was obtained from each patient who participated in the study.

Twelve patients receiving a BSSO advancement were included between December 2020 and March 2021. Patients who participated had to be aged at least 18 years and had to be able to sit upright to complete the measurements. Patients were excluded if they had severe facial deformities, substantial facial hair, previous maxillofacial surgery within the six months before surgery, or a maxillary osteotomy in addition to the BSSO.

Patients received the BSSO according to the method by Obwegeser with Hunsuck modification [14]. The mandible was placed in the planned position with a 3D printed surgical splint. Screw fixation took place with two to four screws per side. Inter-maxillary fixation was secured by rubber bands with the dental braces of the patients. Patients received 12 mg of dexamethasone perioperative and 600 mg ibuprofen three times on the day of the surgery. Patients were allowed to use ibuprofen when needed during the postoperative course. During the follow-up, rubber band fixation was phased out. Both surgery and follow-up were performed by one experienced oral and maxillofacial surgeon.

### 2.2. Measurements

Patients were measured once within the first three weeks postoperatively and once at three months postoperatively. At the first measurement, two 3D photographs were captured within an interval of ten minutes, with the patient leaving the measurement set-up between the two photographs.

Three-dimensional photographs of the head and neck were captured with the 3dMDface^TM^ stereophotogrammetry system (3dMD LCC, Atlanta, GA, USA) by one experienced observer. The 3dMD system consisted of two pods, each equipped with one color and two infrared cameras. This system provided ~190° full-face coverage, meaning that data from ear-to-ear were captured. Before its use, the camera was calibrated to define a 3D coordinate system.

The 3D photographs were taken with the patient sitting, with hips in 90° flexion, the spine vertical, and hands resting on the lap. Adjustments to seating heights were made to include as much of the neck area as possible. For a standardized reproducible head and upper body orientation, the natural head position was used [15]. The midline of the face was aligned towards the camera, and the patients were asked to look straight ahead to a point at eye level on the wall in front of them. To pursue a reproducible position of the jaws, patients were instructed to slowly close the mouth until first occlusional contact and then swallow once. The jaws and facial soft tissues were in a relaxed position while occluding gently. Assessment of the 3D photographs was performed directly after capturing to distillate and retake 3D photographs with large holes, open mouth, or closed eyes.

### 2.3. Data Processing

**Figure 1:**
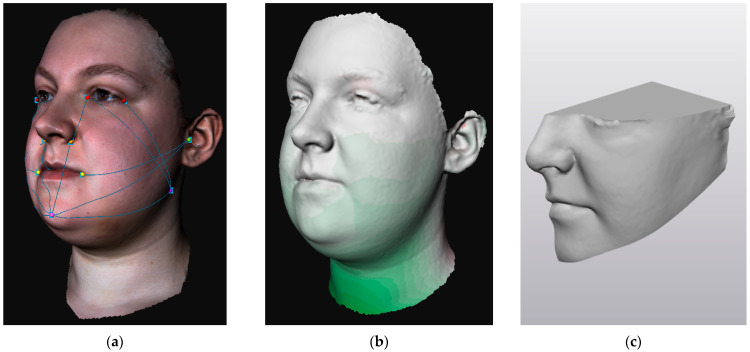
Example of the outcome parameters retrieved from 3D stereophotogrammetry: (**a**) distance measurements of facial categories; (**b**) distance map for obtaining mean and root mean square values; (**c**) closed 3D object of left facial region for obtaining volume difference.

**Figure 2:**
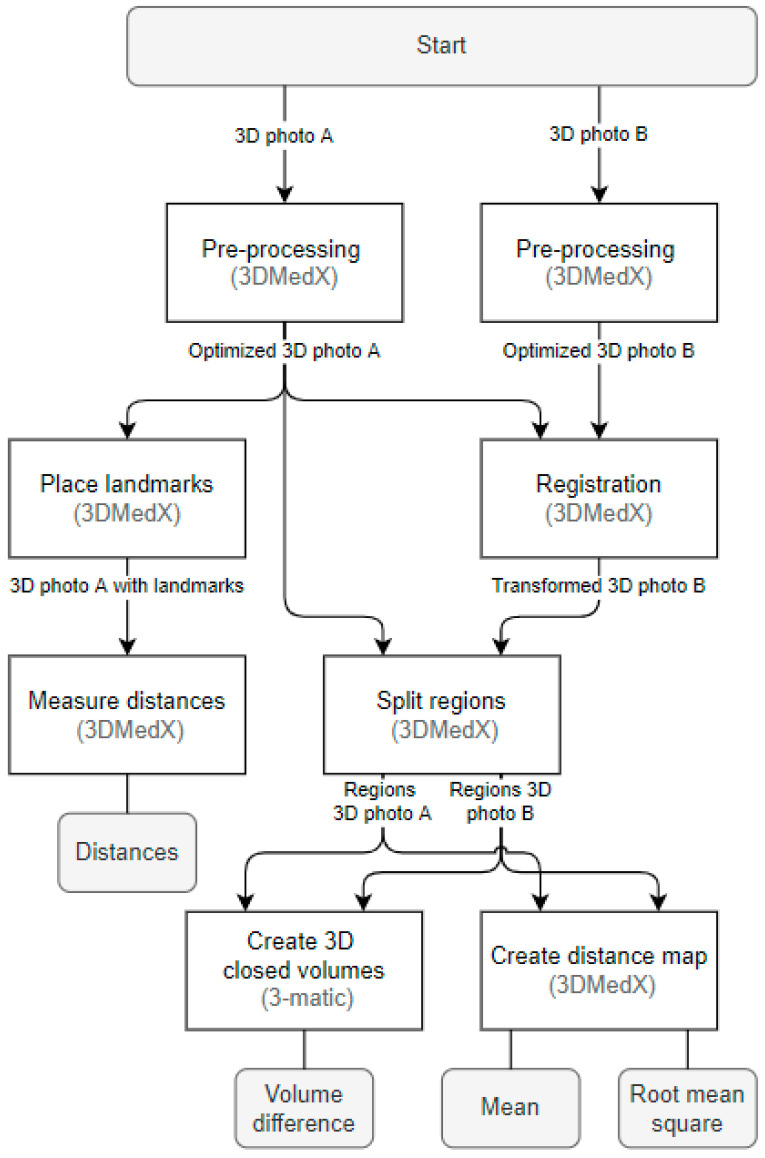
Flowchart for obtaining outcome parameters for 3D stereophotogrammetry.

In order to quantify swelling (1) distances were measured on 3D photographs, (2) distance maps were made between 3D photographs, and (3) volume differences were calculated between 3D photographs (Figure 1). For the distance maps and volume differences, the 3D photograph captured at three months postoperatively was set as the reference since it could be assumed that 80% of the swelling was cleared at that time point [2,9]. The outcome parameters were obtained by processing data in 3DMedX (version 1.2.18.0, Radboudumc, Nijmegen, The Netherlands, https://www.3DMedX.nl, accessed on 1 November 2022) and Materialise 3-Matic (version 15.0, Materialise NV, Leuven, Belgium), as shown in Figure 2.

Before retrieving the outcome parameters, 3D photographs were pre-processed to eliminate irregularities and structural problems. Parts of the 3D photograph with hair and clothes were removed and excluded.

#### 2.3.1. Distance Measurements

Distances on 3D photographs were measured according to the distance measurements of the head and neck lymphedema program at the M.D. Anderson Cancer Center [16]. These measurements included seven facial distances (for each facial side), three neck circumferences, and two head circumferences. Circumference measurements were partially performed for the included parts of the head and neck. To retrieve the distances on 3D photographs, the shortest distance over the surface of the 3D photograph was calculated between facial landmarks. The landmarks with their known precisions are listed in Table 1. Distances were obtained by calculating the shortest distance over the surface of the 3D photograph between landmarks, as presented in Table 2.

#### 2.3.2. Registration and Region Splitting

Registration of the 3D photograph with the reference 3D photograph was performed to allow the calculation of the distance map parameters and volume differences between the two aligned photographs. Registration was performed by the iterative closest point (ICP) algorithm, which iteratively translated and rotated the 3D photograph until the Euclidean distance to the reference 3D photograph was minimized. The ICP registration was based on the forehead and nasal bridge since these regions were expected to be least affected by swelling. The forehead and nasal bridge were manually selected with the 3D photograph placed in the natural head position.

**Figure 3:**
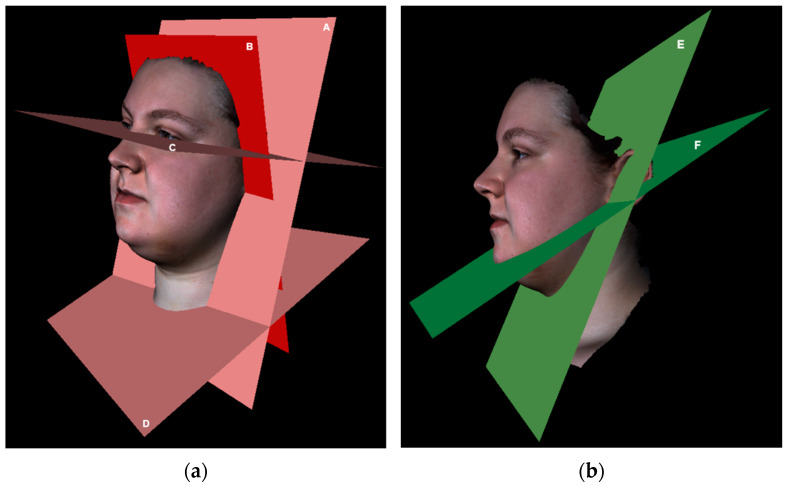
Examples of the planes for splitting 3D photographs into right face, left face, submental, and neck regions. (**a**) A: Neck posterior plane; B: head posterior plane; C: Frankfurter plane; D: neck inferior plane; (**b**) E: face–submental plane; F: submental–neck plane (Mid plane is not shown).

After registration, the two 3D photographs were split into four regions: right face, left face, submental, and neck. Three-dimensional photographs were split by planes, which were defined based on landmarks as presented in Table 3. Landmarks were placed on the reference 3D photograph. The landmarks defining the inferior neck plane were placed on the 3D photograph with the least part of the neck captured. The defined planes were used to split all 3D photographs of the same patient (Figure 3).

#### 2.3.3. Distance Map Parameters

The signed Euclidean distance was calculated from the vertices of each region in a 3D photograph to the closest vertices of the same region in the reference photograph. Visualizing the obtained distances as vertex color resulted in a distance map. The mean and root mean square (RMS) of the distance map of each region were extracted for data analysis. The RMS first squares distances, then takes the mean, and finally neutralizes the squaring by taking the square root. Therefore, the RMS provided more information about the extent of differences between two 3D photographs, while the mean value showed whether there was an overall increase or decrease between the two photographs.

#### 2.3.4. Volume Differences

Volume differences between two 3D photographs were obtained per facial region. For that, each region of the 3D photographs was extruded to the vertical planes (head posterior or neck posterior), which were defined by landmarks on the reference 3D photograph, as shown in Table 3. In some cases, holes remained after extruding because of initial missing parts or inaccuracies in the 3D photograph. These holes were automatically filled by identifying the bad contours in the 3D object and closing the surface within the contour. Visual inspection was performed on any residual holes or peeks, which were manually adjusted when necessary. This semi-automatic method resulted in a closed 3D object for each region in both 3D photographs. Volumes of two corresponding 3D objects were retrieved and subtracted to obtain the volume difference.

#### 2.3.5. Reliability

The reliability of 3D stereophotogrammetry was determined by repeated 3D stereophotogrammetry measurements. Distances were measured on both the test and retest 3D photographs. Moreover, the mean and RMS of the distance map and volume difference were obtained for the test and retest 3D photographs related to the same reference 3D photograph.

### 2.4. Statistical Analysis

All analyses were conducted using Statistical Package for the Social Sciences (SPSS) (version 27, IBM, Chicago, IL, USA). *p*-values below 0.05 were considered statistically significant. A power analysis was conducted, with an expected ICC of at least 0.6. A p_1_ value of 0.9 was chosen; therefore, the sample size had to be at least 11.7 [21].

The reliability was assessed by calculating ICCs between test and retest outcome parameters. ICCs were obtained per distance measurement, per distance category, and for all distance categories together. A two-way mixed model with an absolute agreement and a single measure was applied [22]. ICCs were calculated as $\frac{{\mathrm{MS}}_{R}-{\mathrm{MS}}_{E}}{{\mathrm{MS}}_{R}+\left(k-1\right){\mathrm{MS}}_{E}+\frac{k}{n}\left({\mathrm{MS}}_{C}-{\mathrm{MS}}_{E}\right)}$, in which MS_R_ = mean square for rows, MS_E_ = mean square for error, MS_C_ = mean square for columns, n = number of subjects, and k = number of measurements. The same model was applied to obtain the ICCs for the mean and RMS of the distance map and volume difference. For these parameters, ICCs were obtained for the right face, left face, submental, and neck regions and for all regions together. ICCs were interpreted as poor (<0.50), moderate (0.50–0.75), good (0.76–0.90), and excellent (>0.90) reliability [22]. In addition, the standard error of measurement (SEM) and smallest detectable change (SDC) were calculated for all distances, the mean and RMS of the distance map, and volume difference to indicate the measurement error. Absolute SEM was calculated as $\mathrm{SEM}=\mathrm{SD}\times \sqrt{1-\mathrm{ICC}}$, with SD as the standard deviation of the difference between the test and retest 3D measurements. Absolute SDC was calculated as $\mathrm{SDC}=1.96\times \sqrt{2}\times \mathrm{SEM}$ [23]. The SEM and SDC were also calculated as percentages of the mean of both the test and retest 3D measurements.

## 3. Results

Twelve patients were included in the data analysis, of which there were five males and seven females. The median age was 27 years, with an interquartile range (IQR) of 24 to 48. Median weight, height, and body mass index at the first measurement were, respectively, 87.3 kg (IQR = 70.6–99.3), 183 cm (IQR = 181–191), and 24.7 (IQR = 21.2–28.7) for the males and 65.5 kg (IQR = 57.4–72.7), 173 cm (IQR = 172–175), and 23.1 (19.2–24.3) for the females. Median weight and BMI at the second measurement were, respectively, 85.9 kg (IQR = 71.0–101.4) and 24.3 (IQR = 21.3–29.3) for males and 65.5 kg (IQR = 57.5–75.1) and 23.5 (IQR = 19.2–24.8) for females. All patients were scheduled for a BSSO, with a median advancement of 6 mm (range = 2.5–9.0 mm). No patients were excluded for substantial facial hair because all patients with facial hair needed to shave this before the surgery.

Test-retest reliability was excellent for the overall distances per category from 3D stereophotogrammetry in patients, with ICCs of at least 0.96 (Table 4). The SEM and SDC were smaller than 1% for the overall distances. Individual distance measurements presented often lower ICCs than the overall distances. Nonetheless, all ICCs were still larger than 0.81. The individual distance measurements presented a maximum SEM of 1.50 mm (1.3%) for facial distances and 2.72 mm (0.8%) for circumferences. Maximal SDC was 4.16 mm (3.6%) for facial distances and 7.53 mm (2.2%) for circumferences.

The mean and RMS of the distance map and volume difference from 3D stereophotogrammetry displayed excellent reliability for facial and submental regions, with ICCs larger than 0.92. ICCs for the neck region varied from 0.89 to 0.92. The neck region presented the largest SEM and SDC, with values of 0.34 mm (9.2%) and 0.93 mm (25.6%) for the mean distance map and 5.09 mL (14.8%) and 14.1 mL (41.0%) for the volume difference, respectively.

## 4. Discussion

Three-dimensional stereophotogrammetry is promising for measuring soft tissue changes caused by swelling in the head and neck area. Nonetheless, the reliability for measurements in the lower face and neck areas has not yet been determined [10,11]. This study demonstrated that 3D stereophotogrammetry has an excellent reliability for overall distance measurements, mean and RMS of the distance map, and volume difference in patients after orthognathic surgery.

In our research, the reliability of 3D stereophotogrammetry was excellent for distances per category (right face, left face, head circumferences, and neck circumferences) and good to excellent for individual distances. This somewhat lower reliability for individual distances could be explained by the small sample size since it is known that reliability depends on the sample size [24]. Moreover, the reliability of measured distances depended on the precision of the landmark placement on 3D photography. ICCs were lowest for distances from gonion to alare and gonion to pogonion. Earlier studies reported poor precision for the gonion landmark with more than 3 mm deviation [17,18,19,20], clarifying why distances defined by this landmark presented reduced reliability in our study. In addition, the reliability of the neck circumference measurements may have been restricted due to the limited range of the neck captured with 3D stereophotogrammetry. A posterior neck plane was defined to establish beginning and endpoints for the partial neck circumferences. Due to this clear definition, good-to-excellent reliability was still retrieved for the neck circumferences based on ear-to-ear data. Nevertheless, in some cases, relevant areas of the neck region were missing, which hindered neck circumference measurements.

Individual distance measurements presented a maximal SEM of 1.3%, demonstrating that these measurements can vary up to 1.3% from the true score. The maximal SDC was 3.6% for facial distances and 2.2% for circumferences. These SDC values revealed that the minimal value to measure important changes should be larger than 3.6% for facial measurements and 2.2% for circumference measurements.

The mean and RMS of the distance map and volume difference displayed excellent reliability for the face and submental regions and slightly lower reliability for the neck region. Distance maps and volume differences depended on the reproducibility of the position of the patient. Although the natural head position has been reported as a reproducible head orientation [15], achieving natural head position depended on how well instructions were followed by the patient. Moreover, a reproducible neck position has not yet been standardized. This may explain why reliability was slightly lower for the neck region.

The neck region presented the largest SEM and SDC for the volume difference, with values of 5.09 mL and 14.1 mL, respectively. For the right and left facial regions, SDC was only 1.48 mL and 1.72 mL, respectively. This is somewhat lower than the SDC of 5.9 mL reported by van der Meer et al. [11] for measurements of artificial swelling of the buccal area in healthy subjects.

### 4.1. Strengths and Limitations

Three-dimensional stereophotogrammetry as a measurement instrument for soft tissue swelling had some restrictions. The 3dMDface^TM^ stereophotogrammetry system that was used in this study could only capture ear-to-ear data. Moreover, some areas could not be captured in all patients, for example, the submental and submandibular areas in skinny patients with a strong jawline. On the other hand, in patients with more soft tissue due to a higher BMI or swelling, identification of skeletal landmarks on 3D photographs was more difficult. Data processing was limited to some manual and semi-automatic steps. The selection of the forehead and nasal bridge for registration and identification of landmarks were performed manually. Closed 3D objects could be obtained automatically but still needed a visible check and sometimes additional manipulation. Moreover, a straightforward outcome parameter for the total volume difference, including increases and decreases within one region, was not retrieved; however, distance maps provided a clear and fast overview of the changes that occurred.

### 4.2. Clinical Implications

Three-dimensional stereophotogrammetry demonstrated excellent reliability for overall distance measurements (ICC = 0.998) and somewhat lower reliability for individual distance measurements (ICC ≥ 0.818). The poorer properties for individual distances imply that clinical decisions should not be based on single-distance measurements. Distance map and volume differences from 3D stereophotogrammetry gave additional information for the evaluation of soft tissue swelling. Mean and RMS values of the distance map and volume differences from 3D stereophotogrammetry presented in general excellent reliability (ICC ≥ 0.895). Maximum SEM was 5.09 mL for the neck region. The disparity in neck areas could be the consequence of a difference in position, which emphasizes the need for a standardized neck position for optimal clinical application. Moreover, a 3D stereophotogrammetry system that captures 360° of data may improve the monitoring of edema in the neck region.

### 4.3. Future Research

This study revealed several measurement options in 3D stereophotogrammetry for quantifying soft tissue changes caused by swelling in the head and neck area after orthognathic surgery. Before clinical and scientific application of 3D stereophotogrammetry for quantifying soft tissue changes in patients after orthognathic surgery, the validity, responsiveness, and minimal important change should also be determined. Moreover, the possibilities for 360° 3D stereophotogrammetry should be explored to establish the optimal measurements for submental and neck edema.

## 5. Conclusions

Three-dimensional stereophotogrammetry is promising for the measurement of soft tissue changes caused by swelling in the lower face and neck areas after orthognathic surgery. This study demonstrated that 3D stereophotogrammetry has an excellent reliability for distance measurements and volume difference in patients after orthognathic surgery.

## Figures and Tables

**Table 1:** Facial and neck landmarks with their abbreviations, explanations, and known precisions. Known precision is scored as poor (>3 mm), fair (2–3 mm), moderate (1–2 mm), good (0.5–1 mm), or very good (<0.5 mm).

Landmark	Abbreviation	Explanation	Precision
Alare	A	The most lateral point on the alar contour.	Moderate [17,18] to good [19,20]
Cheilion	Ch	The point located at the labial commissure.	Very good [19,20]
Endocanthion	En	The soft tissue point at the inner commissure of the eye fissure.	Good [20] to very good [17,19]
Exocanthion	Ex	The soft tissue point at the outer commissure of the eye fissure.	Good [19,20] to very good [17,18]
Soft tissue gonion	Go’	The most posterior inferior point of the soft tissue over the angle of the mandible. Can also be constructed by bisecting the angle formed by the intersection of the mandibular plane and ramus of the mandible.	Poor [17,18,19,20]
Soft tissue hyoid	H’	The soft tissue midpoint at the transition between head and neck.	Not available *
Neck inferior	N_inf_	Most inferior point of the (included) neck area, following skin tension lines.	Not available *
Neck middle	N_mid_	The midpoint of the (included) neck area, following skin tension lines.	Not available *
Neck superior	N_sup_	Most superior point of the neck area, following skin tension lines.	Not available *
Otobasion inferior	OBI	The most inferior point on the earlobe, located at the attachment of the ear to the face.	Good [18] to very good [19]
Otobasion superior	OBS	The most superior point of the ear, located at the attachment of the ear to the face.	Not available
Soft tissue pogonion	Pg’	The most anterior midpoint of soft tissue over the mandibular symphysis.	Good [19,20]
Subnasal	Sn	The midpoint on the nasolabial soft tissue contour between the columnella crest and the upper lip.	Good [17,19]
Tragus	T	Cartilage prominence of the external ear, in front of the concha.	Not available

* Newly defined landmarks.

**Table 2:** Distance measurements performed on 3D photographs using facial landmarks (Table 1). Distance measurements 1–7 were performed on the right and left sides of the head, using the landmarks of the corresponding side.

Distance Measurement According to M.D. Anderson Cancer Center [16]		Landmarks on 3D Photograph
1	Tragus to mental protuberance	T to Pg’
2	Tragus to mouth angle	T to Ch
3	Mental protuberance to internal eye corner	Pg via A to En
4	Mandibular angle to external eye corner	Go’ to Ex
5	Mandibular angle to internal eye corner	Go’ to En
6	Mandibular angle to nasal wing	Go’ to A
7	Mandibular angle to metal protuberance	Go’ to Pg’
Head diagonal	Diagonal circumference: chin to crown of the head	Left OBS via Pg’ to right OBS
Head vertical	Vertical circumference: in front of the ear	Left OBS via left Go’, H, and right Go’ to right OBS
Neck superior	Superior neck circumference	Left N N_sup_ via mid N_sup_ to right N_sup_
Neck middle	Middle neck circumference	Left N_mid_ via mid N_mid_ to right N_mid_
Neck inferior	Inferior neck circumference	Left N_inf_ via mid N_inf_ to right N_inf_

**Table 3:** Planes for splitting 3D photographs into right face, left face, submental, and neck regions.

Plane	Defined by	Explanation
Frankfurter	Landmarks: left and right Ex and one OBS of choice.	Part of the 3D photograph superior to this plane is excluded, defining the superior border of the facial regions.
Face–submental	Landmarks: left and right OBI and Pg’.	Splits the face region from the submental region.
Submental–neck	Landmarks: left and right OBI and H’.	Splits the submental region from the neck region.
Neck inferior	Landmarks: left, middle, and right N_inf_.	Part of the 3D photograph inferior to this plane is excluded, defining the inferior border of the neck region.
Mid	Landmarks: Pg’, Sn, and midpoint between left and right Ex.	Splits the face in left and right regions.
Head posterior	Landmarks: left and right OBI and one OBS of choice.	Part of the 3D photograph posterior to this plane is excluded, defining the posterior border of the facial regions.
Neck posterior	Landmarks: left and right OBI; Plane defined by the landmarks: left and right A, and one OBS of choice.	Part of the 3D photograph posterior to this plane is excluded, defining the posterior border of the neck region.

**Table 4:** Intraclass correlation coefficients (ICCs) with 95% confidence intervals (CI), standard error of measurement (SEM), and smallest detectable change (SDC) for test-retest measurements at 3D photographs.

Parameter			ICC (95% CI)	SEM (mm)	SDC
Distances on 3D photograph (mm)	Right face	1	0.915 (0.739–0.975)	0.89 (0.6%)	2.46 (1.6%)
		2	0.922 (0.763–0.977)	0.74 (0.6%)	2.04 (1.7%)
		3	0.929 (0.782–0.979)	0.84 (0.7%)	2.32 (2.0%)
		4	0.949 (0.835–0.985)	0.51 (0.5%)	1.41 (1.3%)
		5	0.974 (0.916–0.992)	0.27 (0.2%)	0.74 (0.5%)
		6	0.925 (0.768–0.978)	0.56 (0.5%)	1.56 (1.3%)
		7	0.877 (0.638–0.963)	1.30 (1.1%)	3.59 (3.1%)
		Overall	0.985 (0.977–0.991)	0.32 (0.3%)	0.89 (0.7%)
	Left face	1	0.965 (0.887–0.990)	0.40 (0.3%)	1.12 (0.7%)
		2	0.933 (0.791–0.980)	0.69 (0.6%)	1.91 (1.6%)
		3	0.987 (0.956–0.996)	0.18 (0.2%)	0.50 (0.4%)
		4	0.877 (0.639–0.962)	1.15 (1.1%)	3.18 (3.0%)
		5	0.950 (0.837–0.985)	0.51 (0.4%)	1.40 (1.1%)
		6	0.818 (0.501–0.943)	1.44 (1.2%)	3.99 (3.4%)
		7	0.886 (0.656–0.996)	1.50 (1.3%)	4.16 (3.6%)
		Overall	0.987 (0.957–0.996)	0.33 (0.3%)	0.93 (0.8%)
	Neck circumference	Superior	0.974 (0.912–0.992)	0.77 (0.4%)	2.12 (0.6%)
		Middle	0.936 (0.794–0.981)	2.72 (0.8%)	7.53 (2.2%)
		Inferior	0.978 (0.926–0.994)	0.61 (0.3%)	1.68 (0.8%)
		Overall	0.994 (0.988–0.997)	0.56 (0.2%)	1.55 (0.5%)
	Head circumference	Diagonal	0.975 (0.915–0.993)	0.79 (0.4%)	2.20 (1.2%)
		Vertical	0.952 (0.799–0.987)	1.41 (0.7%)	3.90 (2.1%)
		Overall	0.962 (0.888–0.985)	0.26 (0.1%)	0.71 (0.4%)
	Overall		0.998 (0.998–0.999)	0.19 (0.1%)	0.52 (0.3%)
Mean distance map (mm)	Right face		0.980 (0.903–0.995)	0.02 (2.6%)	0.05 (7.4%)
	Left face		0.987 (0.975–0.996)	0.01 (1.5%)	0.04 (4.3%)
	Submental		0.974 (0.915–0.992)	0.04 (1.4%)	0.10 (4.0%)
	Neck		0.920 (0.722–0.977)	0.34 (9.2%)	0.93 (25.6%)
	Overall		0.950 (0.909–0.972)	0.15 (7.6%)	0.40 (21.1%)
Root mean square distance map	Right face		0.924 (0.761–0.977)	0.07 (5.2%)	0.20 (14.3%)
	Left face		0.974 (0.917–0.922)	0.03 (1.8%)	0.08 (4.9%)
	Submental		0.950 (0.836–0.985)	0.07 (2.4%)	0.20 (6.6%)
	Neck		0.895 (0.679–0.969)	0.53 (6.2%)	1.46 (17.2%)
	Overall		0.971 (0.950–0.984)	0.14 (3.8%)	0.39 (10.7%)
Volume difference (mL)	Right face		0.942 (0.814–0.983)	0.62 (11.7%)	1.72 (32.5%)
	Left face		0.955 (0.851–0.987)	0.53 (5.5%)	1.48 (15.3%)
	Submental		0.979 (0.928–0.994)	0.23 (1.4%)	0.64 (3.8%)
	Neck		0.905 (0.692–0.972)	5.09 (14.8%)	14.1 (41.0%)
	Overall		0.914 (0.853–0.951)	2.65 (16.0%)	7.34 (44.5%)

## Data Availability

Not applicable.
